# Concentration of Polycyclic Aromatic Hydrocarbons and Estimated Human Health Risk of Water Samples Around Atlas Cove, Lagos, Nigeria

**DOI:** 10.5696/2156-9614-8.20.181210

**Published:** 2018-12-06

**Authors:** Oluwafunmilayo O. Olayinka, Adetomi Adeola Adewusi, Olusoji Olujimi Olarenwaju, A.A. Aladesida

**Affiliations:** 1 Department of Environmental Management and Toxicology, Federal University of Agriculture, Abeokuta; 2 Department of Zoology, Federal University of Agriculture, Abeokuta

**Keywords:** PAHs, pollution, LMW, HMW, risk assessment, crude oil

## Abstract

**Background.:**

Polycyclic aromatic hydrocarbons (PAHs) are common pollutants in water and have been reported to cause severe health effects in humans as well as the ecosystem.

**Objectives.:**

This study examined the concentrations of polycyclic aromatic hydrocarbons and estimated the human health risk from water samples around Atlas Cove jetty, Lagos, Nigeria.

**Methods.:**

Physical and chemical parameters and PAHs were determined in Atlas Cove jetty water using standard methods from June to August 2016 at five different points of activity. Chronic daily intake through ingestion, exposure dose via dermal absorption and carcinogenic risks were calculated for children and adults.

**Results.:**

Electrical conductivity values ranged from 23,600±57.74 - 30,000±57.74 μS/cm. Dissolved oxygen ranged from 6.27±0.46 - 9.60±0.00 mg/L. Biochemical oxygen demand levels ranged from 2.93±0.61 - 7.33±0.23 mg/L and total dissolved solid ranged from 17,500±57.74 - 20,000±57.74 mg/L for the water samples, which was higher than permissible limits. The values obtained for pH, chemical oxygen demand, nitrate, sulphate and phosphate for the water samples were within World Health Organization (WHO) limits except for pH at point 2 (3.18±0.02). A total of eleven PAH congeners were detected in the water samples. The concentrations of total PAHs observed in water samples ranged from 46 - 507 μg/L. Low molecular weight PAHs were more dominant in all samples. It was observed that 2–3 ring PAHs accounted for 63.64% of PAHs, 4-rings PAHs accounted for 27.27% of PAHs, and 5–6 ring PAHs accounted for 9.09% of PAHs.

**Conclusions.:**

Carcinogenic risks calculated for both adults and children were higher than the United States Environmental Protection Agency (USEPA) acceptable cancer risk, and much higher for children, which suggests that children could be prone to cancer through ingestion. Fauna and flora around the Atlas Cove jetty may be at risk due to water pollution.

**Competing Interests.:**

The authors declare no competing financial interests.

## Introduction

Polycyclic aromatic hydrocarbons (PAHs) are organic chemical compounds that can occur naturally in the environment, but their occurrence can be accelerated by anthropogenic activities. They can be biologically amplified to high concentrations in food webs.^1^ Levels of PAHs classified as priority organic pollutants have steadily increased in recent years and occur in food, air, water, soil and sediments.^2^,^3^ The main sources of these contaminants in the environment include forest fire, natural petroleum seeps, combustion of fossil fuel, coal burning, industrial and municipal waste, waste water and sewage. They are among the most widespread persistent organic pollutants in the water environment. The solubility of PAHs decreases in water with increasing molecular weight, resulting in low concentrations in the water column.^4^,^5^ Due to their hydrophobicity, the presence of PAHs in surface or ground water indicates pollution. Some PAHs occur in the environment at low concentrations due to their low biodegradability and persist with elimination difficulties. Consequently, low molecular weight (LMW) PAHs have a short residence time in the water column due to volatilization, oxidization and can quickly be eliminated.^6^ High molecular weight (HMW) PAHs are readily adsorbed on particles in the surface water with the hydrophobic elements readily attaching to bottom sediments.^7^ The types of PAHs present in water provide information on the derivative sources of organic contaminants. The presence of LMW PAHs such as naphthalene, fluorine and acenaphthene in environmental media indicate natural or petrogenic PAH contamination, while a prominent concentration of HMW PAHs (fluoranthene, phenanthrene and pyrene) and fewer LMW PAHs implies combustion or pyrolytic origins. Polycyclic aromatic hydrocarbons are toxic, carcinogenic and mutagenic to all organisms, including humans. They can also cause reproductive abnormalities, immune suppression and developmental toxicity.^8^

Access to potable water is an important element required for sustainable development. Adequate sanitation and increased water for food production and industry contribute to health, livelihood and improved economic development.^9^ Water bodies are primary reservoirs of various forms of pollutants, from both natural and anthropogenic sources. These pollutants are introduced by increased industrialization, technological development, a growing human population, oil exploration and exploitation, agricultural and domestic waste run-off.^10^ Oil spillage enters into environment through various methods such as leaking pipes, resulting in pollution.

Oil spillage has led to contamination of aquatic and terrestrial environments. In very large quantities, it could disrupt the entire ecosystem of the area, leading to the death of aquatic organisms and gross contamination of water, making water unfit for other domestic and industrial purposes. Water pollution has both short- and long-term effects. Habitat destruction, loss of biodiversity and water pollution have important implications for the local population. Oil spillage and petroleum products are anthropogenic sources of PAHs in the environment.^11^ Organic pollutants such as PAHs have a tendency to accumulate in biota and undergo food chain magnification.^12^ The Atlas Cove jetty, which serves as a major storage depot for imported petrol before distribution, increases the possibility of contamination during the offloading process from vessels into the Atlas Cove product storage tank. Its environs also serve as a shipping route with heavy vessel traffic. The presence of PAHs in water indicates chronic pollution, and therefore, there is a need to evaluate water quality to ensure the safety of aquatic biota. The objective of the present study was to determine the physical and chemical properties and concentrations of PAHs in water from Atlas Cove and to estimate the consequent human health risks.

**Abbreviations**
*BaA*: Benzo(a)anthracene
*BaP*: Benzo(a)pyrene
*BaPeq*: Benzo(a)pyrene equivalents
*BOD*: Biochemical oxygen demand
*DO*: Dissolved oxygen
*EC*: Electrical conductivity
*HMW*: High molecular weight
*LMW*: Low molecular weight
*PAHs*: Polycyclic aromatic hydrocarbons
*TDS*: Total dissolved solids
*USEPA*: United States Environmental Protection Agency
*WHO*: World Health Organization

## Methods

**Figure 1:**
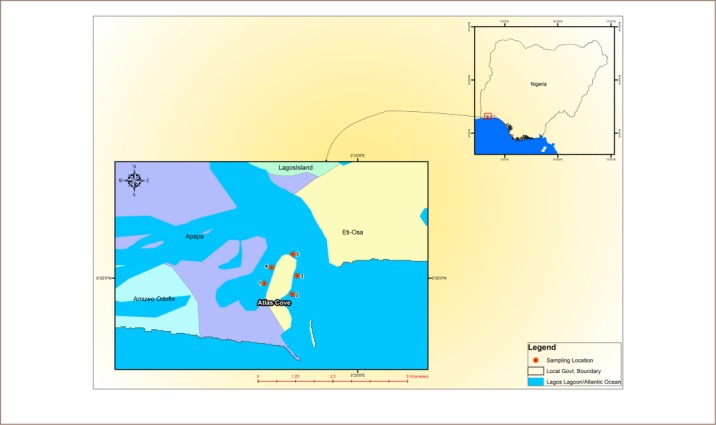
Map of study area showing sampling points

The study was carried out around the Atlas Cove jetty (*Figure 1*) on the Commodore Channel in Lagos State. Atlas Cove is an offloading and storage depot where oil is stored and distributed and experiences frequent oil spills into the surrounding environment. Lagos State is located between latitudes 6° 21′ N and 6° 34′ N and longitudes 3° 01′ E and 3° 27′ E, in the southwestern region of Nigeria and is bounded in the north and east by Ogun State, south by the Atlantic Ocean, and to the west by the Benin Republic. The water around Atlas Cove is brackish, i.e. a mixture of fresh and marine water. The Lagos Lagoon forms part of an extensive water system made up of lagoons and creeks found along the coast line of Nigeria, Benin Republic, Togo and Cote d'Ivoire. It is connected to the Atlantic Ocean by Commodore Channel (on which the jetty is situated) in Lagos. The lagoon is located between longitudes 3° 23′ and 3° 40′ E and latitudes 6° 22′ and 6° 38′ N. The lagoon plays an important role in the surrounding community due to the prosperous molluscan and fishing exploitation and commerce. It is also known for heavy traffic of water vessels. Urban and industrial wastes and oil spills are a major source of pollutants in the lagoon. A large population depends on the lagoon as source of potable water and recreational activity.

### Sampling

Triplicate samples were collected at 5 points between June and August of 2016. Samples were collected in the morning using pre-washed bottles rinsed with the water sample and dipped around 15 cm below the water level at designated sampling points. Samples were then stored in an ice chest. Samples for PAHs analysis were collected in amber bottles.

Water samples were stored at 4°C in the fridge prior to analysis. Physical and chemical water parameters such as electrical conductivity (EC), pH, temperature, total dissolved solids (TDS), dissolved oxygen (DO), salinity, biochemical oxygen demand (BOD), nitrate, phosphate, alkalinity, chemical oxygen demand (COD) and sulphate were determined according to the American Public Health Association.^13^

### Reference standards

A PAH standard mix solution of 16 United States Environmental Protection Agency (USEPA) priority PAHs, each at 100 μg/L in dichloromethane, were purchased from Sigma – Aldrich (St. Louis, MO, USA). The surrogate standard was a mixture containing naphthalene −d_8_ (N-d_8_), acenaphthene-d_10_ (Ace-d_10_), phenanthrene-d_10_ (P-d_10_), chrysene-d_12_ (Ch-d_12_) and perylene-d_12_ (Per-d_12_), which was added to the samples before extraction and used as an internal standard. Stock solutions were used to prepare working standard solutions for calibration and spiking experiments.

### Extraction procedure for polycyclic aromatic hydrocarbons in water

Polycyclic aromatic hydrocarbons in water samples were extracted according to the method described by Laboratory Analytical Work Instruction.^14^ Next, 500 ml of water sample (unfiltered) was poured into a separating flask. Then, 40 ml of dichloromethane (DCM) was added into the flask and shaken, with pressure released at intervals. The sample was allowed to stand until two distinct layers were formed in the flask. The lower layer (organic extract) of the sample was filtered into a beaker through a filter paper containing glass wool and anhydrous sodium sulphate. The process was repeated two times with 20 ml of the extracting solvent, dichloromethane DCM, added. The extracts were combined and concentrated by evaporation at room temperature overnight in a fume cupboard by covering with perforated aluminum foil. Sample clean-up was done by USEPA Method 3630C.^15^

A 600 × 19 mm clean up column was prepared. The hole was blocked with glass wool, 3 g of activated silica gel (60 mesh) was added and the column was topped with sodium sulfate Na_2_SO_4_. The column was rinsed by eluting with 20 ml n-hexane and discarded. The concentrated extract was loaded onto the prepared column and eluted with 50 ml n-hexane. The eluates were then concentrated to 1 ml using a rotary evaporator under a gentle stream of pure nitrogen. Then 1 ml of the extract was transferred into a well labeled vial and stored at 4°C prior to gas chromatograph mass spectrometry (GC-MS) analysis.

### Instrumental and analytical conditions

Analyses of PAHs were performed using gas chromatograph mass spectrometry GC-MS in selected ion monitoring (Shimadzu QP 2010 gas chromatograph mass spectrometry equipped with AOC 5000 auto injector). The column used in the present study was a Varian Factor Four fused silica capillary (30 m × 0.25 mm × 0.25 μm film thickness) used for separating target analytes. Helium was used as the carrier gas at a flow rate of 1.2 mL/min. The sample injector temperature was set at 250°C and 300°C, respectively, and samples were injected at a volume of 1 mL in splitless mode. The column initial temperature was 60°C held for 1 minute and ramped from 60°C to 200°C at 10°C/min, held for 2 minutes, and finally to 300°C at 10°C/min and held for 6 minutes. The mass spectrometry conditions were set as follows: ionization source: electron ionization at − 70 eV: ion source temperature: 200°C: store mass range m/z 47–400 μm. Identification of individual PAHs was based on comparison of retention time between samples and standard solutions.

### Quality control

Spiked blank, reagent blank and appropriate PAH standard solutions were included with each set of samples to ensure the integrity of the analytical method and corresponding analytical results. Samples were spiked with 1 μL of 100 mg/L standard mixture consisting of 16 PAHs to 500 mL pre–extracted water samples. Distilled water (500 mL) was first pre-extracted in triplicate with 30 mL dichloromethane as a blank sample. Spiked samples were then extracted and analyzed. There were no target compounds detected in the procedural and solvent blank. Recovery yields were 70–95% and limit of detection for individual PAHs ranged from 0.90 to 140 μg/L with a signal to noise ratio of 3 and limit of quantization of signal to noise ratio of 10.

### Human health risk assessment

For the exposure assessment, the concentrations of different PAHs were converted into their benzo(a)pyrene (BaP) equivalents (BaPeq) for exposure assessment. Benzo(a)pyrene equivalent concentrations were calculated by multiplying the concentrations of carcinogenic PAHs with their corresponding BaP – relative potency equivalency factors for the two PAHs [benzo (a) anthracene, (BaA) and indeno-1, 2, 3-cd pyrene (IcdP)] detected out of the seven PAHs given by the USEPA.^16^

The two pathways of water exposure considered in the present analysis were direct ingestion and dermal absorption, which were calculated by Equations 1 and 2, respectively. These equations were adopted from the USEPA.^17^

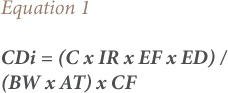
Where, CDi is the chronic daily intake through ingestion (mg/kg day); C is the BaPeq concentration in water (μg/L); IR is the ingestion rate for children (1 L/day) and adults (2 L/day); EF is the exposure frequency (365 days/year); ED is the exposure duration (70 years); BW (kg) is the average body weight for adults (70 kg) and children (15 kg); AT is the average time for carcinogens (ED × 365 days) i.e. 70×365= 25, 550 days; and CF is the conversion factor and is 10^−3^ (USEPA).^16–19^

For dermal absorption;

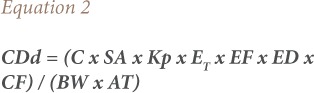
Where, CDd (mg/kg day) is the exposure dose via dermal absorption; C (μg/L) is the concentration of pollutants in water; EF is the exposure frequency and 350 days/year for dermal absorption was used in the calculation; ED is the exposure duration (70 years); BW (kg) is the average body weight for adults (70 kg) and children (15 kg); and AT is the average time for carcinogens (ED × 365 days) i.e. 70×365= 25, 550 days. SA (cm^2^) is the exposed skin area: (adults: 18,000 cm^2^; children: 6,600 cm^2^); Kp (cm/h) is the dermal permeability coefficient 1.2; ET (h/day) is the exposure time for shower and bathing (adults: 0.25 h/day; children: 0.33 h/day) and CF is the conversion factor 10^−3^ (USEPA).^19–22^ SF is the cancer slope factor which is expressed as oral administrative dose derived from rodent feeding studies, whereas dermal exposure is presented as absorbed dose. According to the Integrated Risk Information System of the USEPA, the cancer slope factor (SF) of BaP is 7.3 (mg/kg/day)^−1^.^18^ Therefore, the SF value for dermal exposure was adjusted with the gastrointestinal absorption adjustment factor (AAF) (USEPA).^17^ The estimation of the gastrointestinal absorption of BaP is 92% in dose-response studies from which the cancer SF for BaP was derived.^23^ Therefore, the SF for dermal BaP exposure is equal to 7.3 (mg/kg/day)^−1^/92% = 7.9 (mg/kg/day)^−1^.

The carcinogenic risks (CRs) of ingestion and dermal exposure were calculated using Equations 3 and 4 adopted from the USEPA.^19^


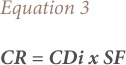



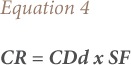


The CR is the probability of developing cancer over a lifetime as a result of exposure to a contaminant. The CDi and CDd are the chronic exposures through ingestion and dermal absorption and SF is the corresponding slope factor. The total carcinogenic risk of BaP in water was calculated as the sum of the CRs from ingestion and dermal exposures.

### Statistical analysis

Results obtained from all samples were subjected to descriptive (mean and standard deviation) and inferential (analysis of variance) statistics and P<0.05 was considered to indicate a significant difference. Means were separated using Duncan's multiple range test.

## Results

The mean values of the physical and chemical properties of water samples around Atlas Cove are presented in Table 1. The results of mean pH values were 6.30±0.01, 3.18±0.02, 6.33±0.01, and 6.77±0.06 and 6.85±0.01 for sampling points 1 – 5, respectively. In all water samples, the highest pH value of 6.85 was obtained at sampling point 5 and lowest value of 3.18 was obtained at sampling point 2. The mean temperature ranged from 26°C to 28.23°C. The highest temperature value (28.23°C) was observed at sampling point 3 and lowest value (26°C) at point 5. The mean values obtained for EC in μS/cm were 23,600±57.74, 25,500±0.00, 27,200±57.74, 30,000±57.74 and 29,900±57.74 for sampling points 1, 2, 3, 4 and 5, respectively. The TDS values in the present study ranged from 17,500±57 to 20,000±57 mgL^−1^. Salinity values varied between 1.39 to 1.7%. Dissolved oxygen values ranged from 6.27±0.46 to 9.60 mgL^−1^ for all sampling points. The highest (7.33± 0.23) and lowest (2.93±0.61) biological oxygen demand values were recorded at sampling points 5 and 1, respectively. The chemical oxygen demand in the present study ranged from 6.74±0.42 to 19.23±0.49 mgL^−1^. The mean values for alkalinity in mgL^−1^ were: 49.33±1.15, 53.33±1.72, 62.67±2.31, 95.33±1.15 and 80.00±2.00 for sampling points 1 to 5, respectively. Mean values of nitrate varied between 11.90 ± 0.08 - 30.07 ± 0.02 mgL^−1^. The highest (1.91± 0.08) phosphate value was recorded at sampling point 1 and lowest (0.27 ± 0.00) at sampling point 5. Sulphate values ranged from 10.17 ± 0.59 to 61.44 ±1.36 for all sampling points.

**Table 1:**
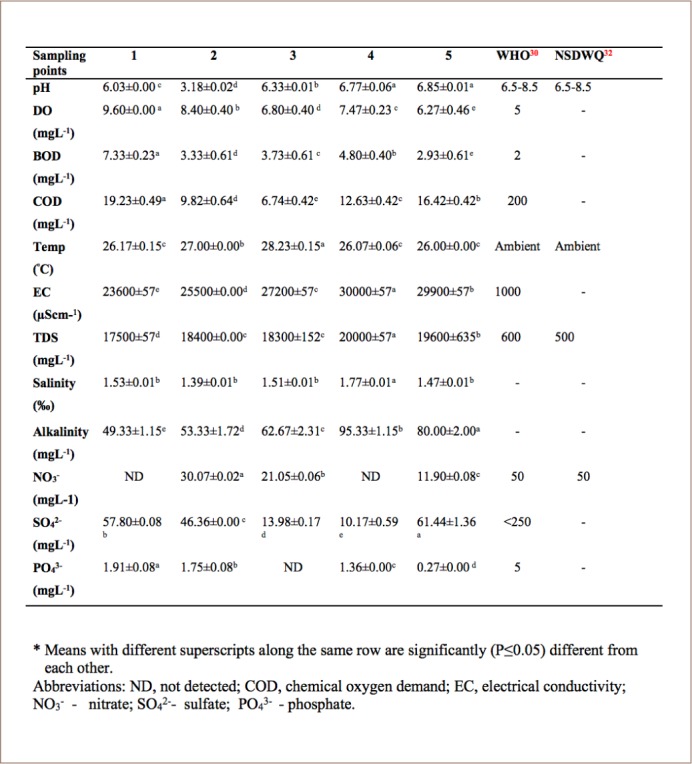
Physical and Chemical Properties of Water Samples

### Concentration of polycyclic aromatic hydrocarbons in water

Concentrations of total PAHs in water samples are shown in Table 2. The concentration of total PAHs ranged from 46 to 507 μg/L. Polycyclic aromatic hydrocarbons from point 2 (507 μg/L) showed the highest concentration, while point 1 had the lowest.

**Table 2:**
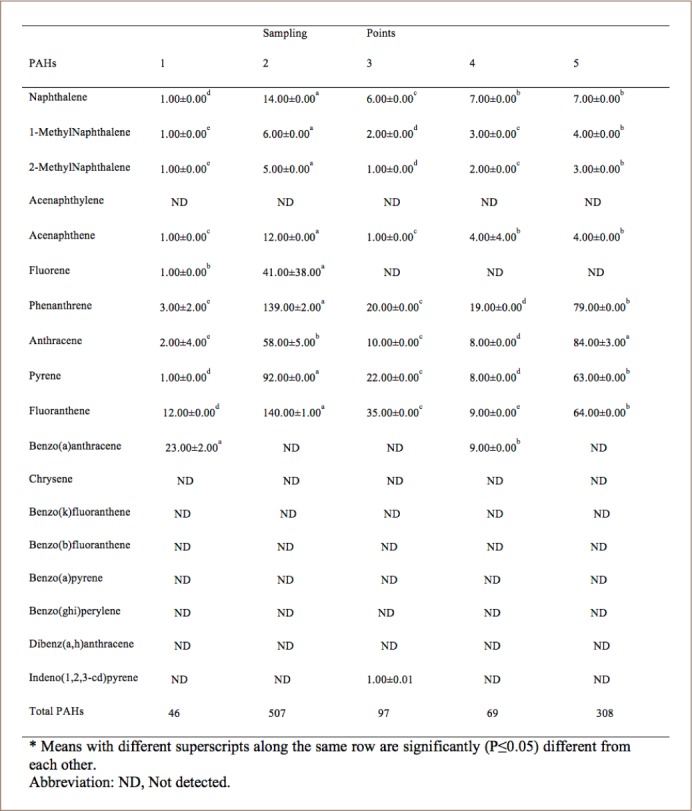
Mean Concentrations of Polycyclic Aromatic Hydrocarbons in Water (μg/L)

## Discussion

The mean values of the physical and chemical properties of water samples around Atlas Cove are presented in Table 1.

The mean pH value showed a trend of slight acidity except for point 2 (3.18±0.02). The importance of water pH cannot be over-emphasized as it helps to regulate metal solubility, water hardness and serves as an indicator of water pollution.^24^ Water quality in an aquatic environment is very important for the survival of its flora and fauna. Aquatic organisms can be affected by water pH as their metabolic activities are pH dependent.^25^ A strong relationship exists between pH and the physiology of most aquatic organisms.^26^ In 2014, Uyom reported that exceptionally high or low pH values in surface waters can be harmful to aquatic organisms.^27^ The pH of the water samples may be attributed to anthropogenic activities around the area and the water may serve as a sink for various wastes. A significant (P≤0.05) difference was observed between the mean pH values for each sampling point. A pH range from 6.09 - 8.45 is ideal for supporting aquatic life, including fish, and Emuedo reported a pH ranging from 6.00 to 9.00 to be optimal for fish production.^28^,^29^ The acid and alkaline death points were reported to be 4.0 and 11.0, respectively, in a study by Olatayo.^26^ However, most values were within the acceptable limits of the World Health Organization (WHO) except for sampling point 2 (3.18±0.02).^30^ The result obtained was similar to the findings of Oketoki who studied a part of the Lagos lagoon.^31^ Temperatures obtained in the present study were within the limits (27°C and 28°C) for river water.^30^,^32^ The temperatures in the present study may be due to an abrupt decrease in temperature where the sea impact is felt during high tide when the cooler sea water penetrates the lagoon or with increased solar radiation.

The EC values reported in the present study were higher than the WHO maximum contaminant level of 1,000 μScm^−1^.^30^ EC is the measure of the capacity of a substance or solution to conduct electric current and is dependent on the amount of dissolved solids present in the water. High EC values indicate the presence of inorganic ions such as calcium (Ca^2+^), magnesium (Mg^2+^), bicarbonate (HCO_3−_), carbonate (CO_3_^2−^), nitrate (NO_3−_) and phosphate (PO_4_^3−^) in increasing concentrations in the water. Total dissolved solids are generally associated with inorganic salt and there is a linear relationship between TDS and conductivity; high conductivity indicates high TDS. It is the measure of the sum of all organic and inorganic substances in a liquid, in molecular, ionized or micro – granular colloidal suspended form. The high TDS concentration is likely an indication of the influx of sea water mixing with the water of the channel. High levels of TDS resulting from dissolved salts may have a profound effect on the various aquatic biota, such as dehydration of fish skin. The TDS level reported in the present study exceeded the WHO maximum contaminant levels of 600 mgL^−1^.^30^

The salinity values showed an indication of brackish habitat and low values reported for salinity may be due to the constant influx of fresh water into the area.^33^ High salinity decreases the exposure of PAHs, while low salinity increases exposure. The DO values obtained may be due to increased aeration resulting from low temperature and high humidity around the study area.^34^ Dissolved oxygen is one of the most important parameters in determining water quality as it is a vital gas for aquatic organisms which depend on water temperature.^35^ When dissolved oxygen falls within the acceptable limit for good water quality, it helps to oxidize and convert poisonous compounds to useful material.^36^ Dissolved oxygen is consumed in water for the decomposition of organic matter, it also encourages feeding, food utilization and high density of fish eggs, larvae and adults within the area. Dissolved oxygen values above 5 mgL^−1^ are considered supportive of marine life, and lower levels are potentially harmful.^28^ At a DO level of 3 mgL^−1^, bottom fishes may start to leave the area, and the growth of sensitive species such as crab larvae is reduced. At 2.5 mgL^−1^, the larvae of less sensitive species of crustaceans may start to die, and the growth of crab species is severely limited. Very high DO concentrations (>20 mgL^−1^) have been reported to be toxic to fish and can cause physiological dysfunction and developmental abnormalities in fertilized eggs and larvae.^26^ The lowest and highest BOD values were recorded at sampling points 5 and 1, respectively. Biological oxygen demand is the amount of oxygen required by aerobic microorganisms to stabilize the organic material of wastewater and is used to indicate the organic strength of water. It indicates the concentration of biodegradable organic matter in water.^37^ When BOD is less than 4 mgL^−1^, water is deemed to be reasonably clean and unpolluted, while a BOD level greater than 10 mgL^−1^ indicates pollution.^38^,^39^

Chemical oxygen demand values in the present study were below the WHO recommended value of 200 mgL^−1^.^30^ Chemical oxygen demand is a measure of organic contamination in water. It is the amount of dissolved oxygen required to cause chemical oxidation of the organic material in water and is a key indicator of the environmental health of surface water.^40^ Chemical oxygen demand a measure of both organic and inorganic agents competing for DO in lake water.^41^ High chemical oxygen demand COD values indicate pollution due to oxidizable organic matter.^42^ The alkalinity value in water indicates the presence of natural mineral salts. The values obtained for alkalinity in the present study were within the acceptable value of 20–200 mgL^−1^. The main species that contribute to alkalinity include bicarbonates, hydroxides, phosphates and borates.^43^ High alkalinity values have a greater resistance to changes in pH. Mean concentrations of nitrate were below the WHO tolerance limits of 50 mgL^−1^. A high concentration of nitrates in water might lead to methemoglobinemia (blue baby syndrome) in humans and eutrophication.^44^

The phosphate values recorded in the present study were below the WHO permissible limit of < 5 mgL^−1^.^30^ Sulphate values obtained in this study varied between the sampling sites. Sampling point 5 had the highest value. Sulphate values recorded in this study were below the WHO permissible limit of 250 mgL^−1^.^30^ Sulphate is generally considered to be non-toxic, however, drinking water containing high amounts of magnesium sulphate or sodium sulphate may lead to intestinal discomfort, diarrhea and consequent dehydration, especially in drinking water containing >500 mgL^−1^ of sulphate.

### Concentration of polycyclic aromatic hydrocarbons in water

The concentrations of total PAHs in water samples are shown in Table 2. Eleven of the sixteen listed as priority pollutants by the USEPA were detected in the water samples. Naphthalene, acenaphthene, fluorene, phenanthrene, anthracene, pyrene, fluoranthene and benzo(a) anthracene were detected in water samples, while chrysene to indeno-1,2,3-cd pyrene were not detected in any sampling points except for point 3. These concentrations were much higher than the WHO limit of 50 ngL^−1^ for surface and coastal waters. Of the total concentrations of PAHs detected in water in three lagoons in Accra reported in a previous study, Chemu lagoon had the highest total concentration of 61.712 μgL^−1^ followed by Korle Lagoon and Kpeshie Lagoon with total PAHs of 38.889 μgL^−1^ and 34.090 μgL^−1^, respectively, which were below the total concentrations obtained in the present study.^45^ The total concentration (72.87 μgmL^−1^) of PAHs detected in Lagos Lagoon was within the range obtained in the present study.^46^ Higher concentrations of PAHs in river water (13.174–26.382 mgL^−1^) were reported which were far higher than the concentrations obtained in the present study.^47^ It was observed that LMW PAHs were more predominant in the samples, similar to the findings reported on the detection of PAHs in drinking water from a large mixed-use reservoir in China.^48^ The presence of LMW PAHs in the water can be attributed to their high vapor pressure and water solubility, while the low concentration or absence of HMW PAHs can be attributed to their lower water solubility and great tendency to adsorb onto solid phases.^49^ Generally, the composition of PAHs is characterized by the greater number of aromatic rings with high carcinogenicity.^50^ The occurrence of PAHs in water based on the compositional patterns (that is, the number of aromatic rings) of PAHs can be classified into low (2–3 ring), intermediate (4-ring) and high (5–6 ring) PAHs.^50^ In this study, the percentage composition of PAHs detected in the samples according to the number of rings are as follows; 2–3-ring PAHs accounted for 63.64% of total PAHs, 4-ring PAHs accounted for 27.27% of total PAHs, while 5–6-ring PAHs accounted for 9.09% of total PAHs detected in water. High molecular weight PAHs contain 4–6 aromatic rings which are not readily bio-degraded by indigenous microorganisms; hence they can persist in the aqueous environment by bioaccumulation in aquatic organisms like fish, crabs and shrimps and pose a greater carcinogenic risk.^51^ The LMW PAHs consist of 2–3 aromatic rings and although they are less carcinogenic, they may also have a toxic effect on many aquatic organisms.^52^ The PAH composition of water and sediments can provide information about their sources. Larger concentrations of LMW PAHs (e.g. acenaphthene, fluorene) in environmental media indicate naturally occurring PAHs (petrogenic and biogenic origins), while PAHs from combustion processes (pyrolytic origin) suggest elevated concentrations of HMW (e.g. phenanthrene, fluoranthene, pyrene) and fewer LMW PAHs. In the present study, it was observed that LMW PAHs occurred in a higher ratio compared to HMW PAHs (7:4), suggesting that PAHs in water samples were generally of natural origin (petrogenic and biogenic) than of anthropogenic origin. Benzo (a) pyrene, which is a marker for the occurrence and carcinogenic effect of PAHs, was not detected in any of the water samples. However, benzo (a) anthracene and indeno-1,2,3-cd pyrene were detected. Sources of PAHs in the coastal environment are petrogenic (if the source is derived from petroleum, e.g. natural oil seepage and oil spills) or pyrogenic (if the source is derived from the incomplete combustion of organic matter and fossil fuel). Polycyclic aromatic hydrocarbons are one of the first and largest set of compounds that have been observed to be strongly mutagenic to laboratory animals and humans and many studies have suggested a link between PAHs exposure and incidences of immune-toxicities and cancers.^50^ A comparison of total PAHs obtained in the present study and different parts of Africa is presented in Table 3.

**Table 3:**
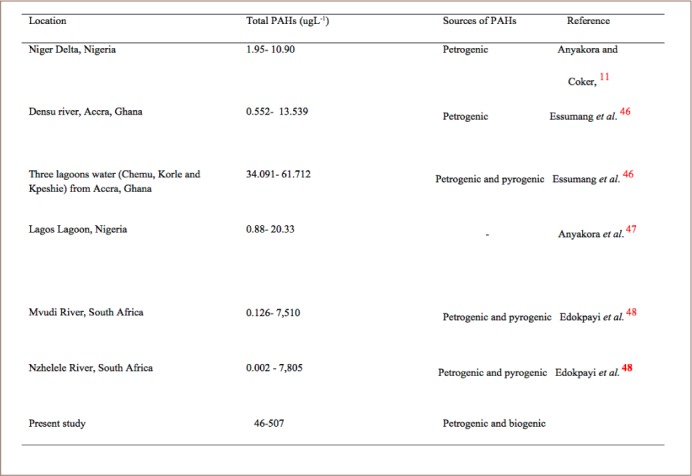
Total Concentrations of PAHs in Water Across Different Locations in Africa

### Health risk assessment

Table 4 also shows the cancer risk for adults and children present in water. Daily BaPeq exposure doses through ingestion and dermal absorption as well as their carcinogenic risk were calculated. For ingestion, the daily BaPeq intake of BaA in adults was within the USEPA acceptable cancer risk of 1×10^−6^ – 10^−4^, while in children it was slightly higher than the upper limit of the acceptable cancer risk. The daily BaPeq intakes of indeno-1, 2, 3-cd pyrene for both adults and children were slightly higher than the USEPA acceptable cancer risk. The carcinogenic risk calculated for both adults and children was higher than the USEPA acceptable cancer risk, but much higher in children, indicating that children may be prone to cancer through ingestion. For dermal absorption, daily BaPeq exposure of BaA and indeno-1, 2, 3-cd pyrene for both adults (2.37×10^−1^ and 7.40×10^−3^, respectively) and children (5.35×10^−1^ and 1.67 ×10^−2^, respectively) showed a risk greater than 1×10^−4^. The carcinogenic risks calculated for dermal exposure for both adults and children were 1.87 and 4.23, respectively. However, the risk for children was 2.5 fold-higher than for adults, which implies that children are more susceptible to skin cancer through dermal exposure. The cancer risk values reported for BaA (2.81×10^−8^ and 1.61×10^−7^) and indeno-1,2,3-cd pyrene (5.40×10^−8^ and 5.23×10^−7^) for ingestion and dermal exposure, respectively, were below the values obtained in the present study.^48^

**Table 4:**
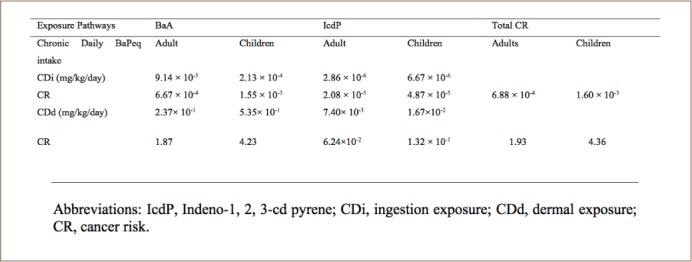
Carcinogenic Risk for Adults and Children

## Conclusions

The physical and chemical parameters in the present study were within permissible limits except for EC, DO, BOD and TDS. The high ratio of LMW PAHs compared to HMW PAHs suggests that PAH contamination around Atlas Cove jetty may be of natural origin (petrogenic and biogenic) or a result of oil spillage. The concentrations of total PAHs were relatively low in water samples due to volatilization and oxidation. The total carcinogenic risk of PAHs in water was higher than the acceptable level.

The results in the present study suggest that dermal exposure of PAHs in water sources poses a greater threat to human health than direct water ingestion. The PAH pollution observed in this study may result in disruption of the aquatic ecosystem and bioaccumulation and/or biomagnification in aquatic biota. This poses a significant threat to aquatic biota and the human population from water consumption. The results of the present study indicate a need for swift action to be taken in response to oil spillage and to reduce fuel pollution by ships. Finally, remediation measures should be put in place to reduce the adverse effects of dermal exposure in activities such as swimming and bathing in water polluted with PAHs.
